# Supplementary material for: Deducing high-accuracy protein contact-maps from a triplet of coevolutionary matrices through deep residual convolutional networks
Source: PLoS Comput Biol. 2021 Mar 26;17(3):e1008865. doi: 10.1371/journal.pcbi.1008865 (PMC8026059; doi:10.1371/journal.pcbi.1008865)
Supplement: S1 Fig — (a) top-L/5 precision, (b) top-L precision. (PDF) [file pcbi.1008865.s001.pdf]

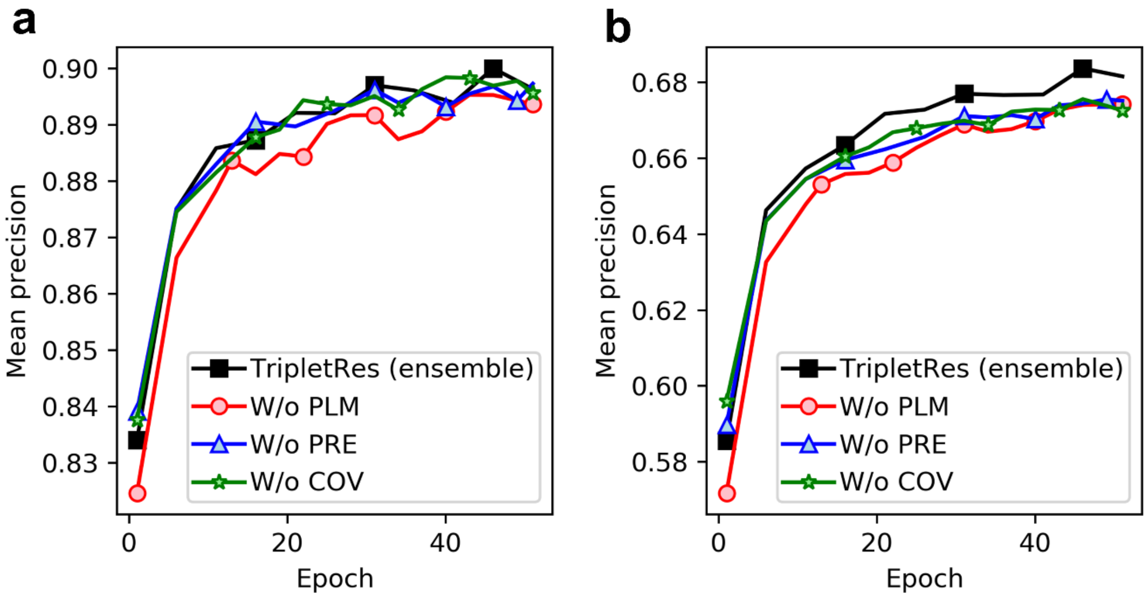

S1 Fig. Comparison of the average precisions over training epochs without individual coevolutionary features and the TripletRes model that ensembles all three sets of features, on the validation set. (a) top-*L*/5 precision, (b) top-*L* precision.
