# Supplementary material for: Deducing high-accuracy protein contact-maps from a triplet of coevolutionary matrices through deep residual convolutional networks
Source: PLoS Comput Biol. 2021 Mar 26;17(3):e1008865. doi: 10.1371/journal.pcbi.1008865 (PMC8026059; doi:10.1371/journal.pcbi.1008865)
Supplement: S2 Fig — (PDF) [file pcbi.1008865.s002.pdf]

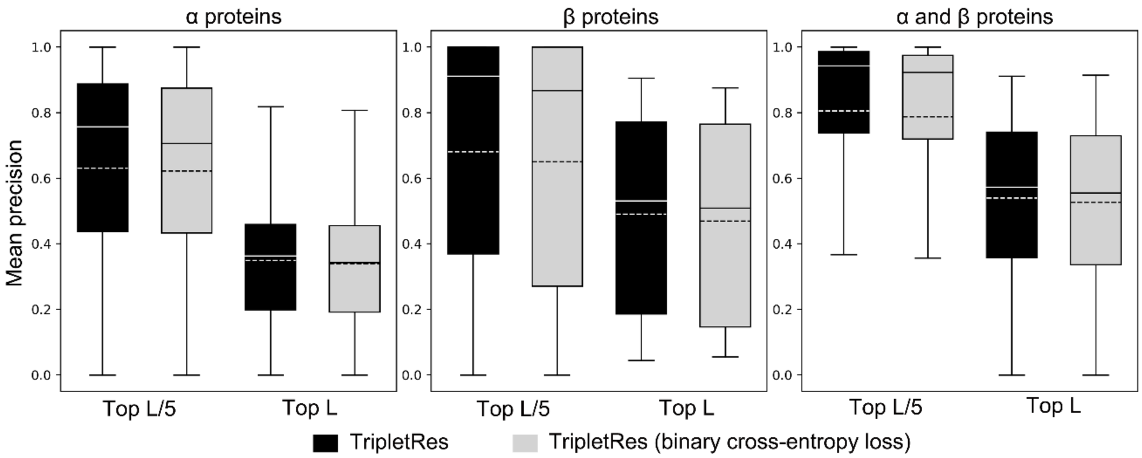

S2 Fig. Comparison of long-range top-$L/5$ and top-L precisions with different loss functions on the different fold types, where median precision and mean precision are marked in solid and dash lines, respectively.
