# Supplementary material for: Deducing high-accuracy protein contact-maps from a triplet of coevolutionary matrices through deep residual convolutional networks
Source: PLoS Comput Biol. 2021 Mar 26;17(3):e1008865. doi: 10.1371/journal.pcbi.1008865 (PMC8026059; doi:10.1371/journal.pcbi.1008865)
Supplement: S3 Fig — (PDF) [file pcbi.1008865.s003.pdf]

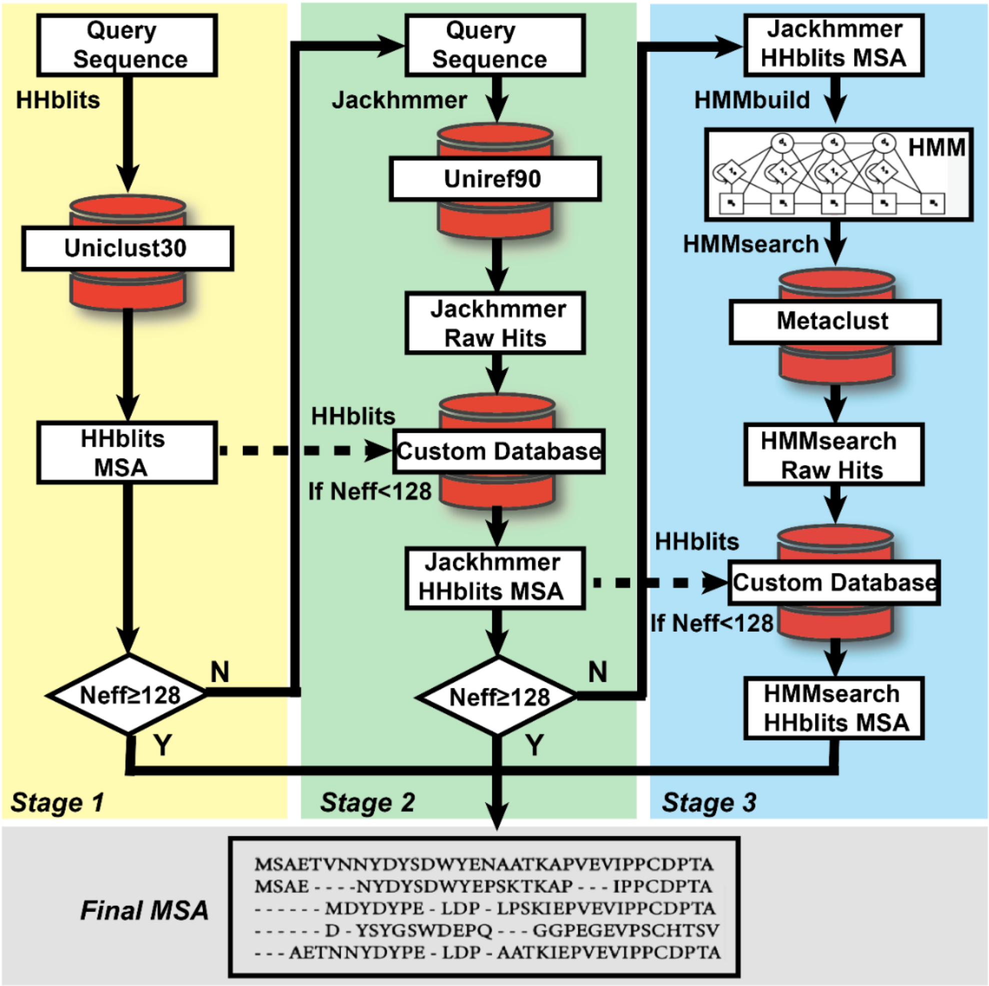

S3 Fig. The DeepMSA pipeline for generating deep multiple sequence alignments for TripletRes. DeepMSA consists of three stages. The query sequence is first searched by HHblits against the Uniclust30 database to generate Stage 1 MSA (yellow background). In Stage 2 (green background), Jackhmmer searches the query sequence through the UniRef90 database to find sequence homologs, which are built into a custom database in HHblits format. HHblits is then used to search Stage 1 MSA through this custom database to get Stage 2 MSA. In Stage 3 (cyan background), the Stage 2 MSA is converted into a hidden Markov model (HMM) by HMMbuild and used by HMMsearch to search the Metaclust metagenome protein sequence database. The identified sequence homologs are reformatted to another HHblits format custom database. The MSA from Stage 2 is then used to search against this custom database to get the final stage alignment. In this incremental MSA construction process, if the MSA from either Stage 1 or Stage 2 reaches Neff>=128, this MSA will be output as final MSA without subsequent stages.
