# Supplementary material for: Deducing high-accuracy protein contact-maps from a triplet of coevolutionary matrices through deep residual convolutional networks
Source: PLoS Comput Biol. 2021 Mar 26;17(3):e1008865. doi: 10.1371/journal.pcbi.1008865 (PMC8026059; doi:10.1371/journal.pcbi.1008865)
Supplement: S1 Table — p-values in parenthesis are from a Student’s t-test between TripletRes and each of the control methods, where bold fonts highlight the best performer in each category. (PDF) [file pcbi.1008865.s004.pdf]

S1 Table. Summary of long-range contact precision by TripletRes and control methods tweaked with DeepMSAs on 50 CASP11&12 FM targets and 195 CAMEO hard targets, sorted in ascending order of top-*L* precision. p-values in parenthesis are from a Student's t-test between TripletRes and each of the control methods, where bold fonts highlight the best performer in each category.

Methods	50 CASP FM targets				195 CAMEO hard targets			
	L/10	*L*/5	*L*/2	*L*	*L*/10	*L*/5	*L*/2	*L*
CCMpred	0.416 (1.0e-11)	0.374 (3.2e-13)	0.264 (2.6e-16)	0.187 (4.5e-17)	0.451 (1.0e-50)	0.411 (5.7e-56)	0.314 (2.8e-66)	0.229 (4.6e-67)
DNCON2	0.599 (3.5e-06)	0.551 (6.8e-06)	0.460 (2.5e-06)	0.353 (8.2e-08)	0.670 (1.4e-13)	0.622 (4.8e-18)	0.503 (2.0e-29)	0.379 (3.6e-36)
MetaPSICOV2	0.571 (2.8e-07)	0.513 (5.5e-08)	0.401 (1.9e-10)	0.299 (1.2e-12)	0.594 (7.0e-24)	0.541 (6.2e-29)	0.431 (3.4e-38)	0.323 (3.8e-42)
DeepContact	0.629 (1.3e-07)	0.583 (1.3e-06)	0.478 (1.3e-07)	0.360 (6.8e-10)	0.699 (2.8e-15)	0.643 (9.3e-21)	0.508 (2.6e-35)	0.384 (1.1e-38)
ResPRE	0.709 (2.9e-03)	0.660 (8.8e-04)	0.549 (1.6e-04)	0.429 (4.2e-05)	0.770 (6.0e-05)	0.725 (1.4e-05)	0.599 (3.8e-12)	0.457 (7.9e-18)
TripletRes	**0.771**	**0.714**	**0.597**	**0.464**	**0.801**	**0.756**	**0.637**	**0.491**
