# Supplementary material for: Deducing high-accuracy protein contact-maps from a triplet of coevolutionary matrices through deep residual convolutional networks
Source: PLoS Comput Biol. 2021 Mar 26;17(3):e1008865. doi: 10.1371/journal.pcbi.1008865 (PMC8026059; doi:10.1371/journal.pcbi.1008865)
Supplement: S2 Table — (PDF) [file pcbi.1008865.s005.pdf]

S2 Table. Summary of long-range contact precision by TripletRes, TripletRes (Post-CASP13) and trRosetta based on the same MSAs on 37 hybrid test sequences.

Methods	*L*/10	*L*/5	*L*/2	*L*
TripletRes	0.775	0.698	0.578	0.434
trRosetta	**0.821**	**0.772**	**0.648**	**0.484**
TripletRes (Post-CASP13)	0.814	0.762	0.623	0.471
