# Supplementary material for: Deducing high-accuracy protein contact-maps from a triplet of coevolutionary matrices through deep residual convolutional networks
Source: PLoS Comput Biol. 2021 Mar 26;17(3):e1008865. doi: 10.1371/journal.pcbi.1008865 (PMC8026059; doi:10.1371/journal.pcbi.1008865)
Supplement: S1 Text — (PDF) [file pcbi.1008865.s006.pdf]

S1 Text. Explanation that DCA models capture linear relationships between residues

Considering the pseudolikelihood maximized Potts model as an example, the marginal probability of l-th position in the sequence is defined by equation (5) in main text:

$$P\left(\sigma_l = \sigma_l^{(m)} \middle| \sigma_{\backslash l} = \sigma_{\backslash l}^{(m)}\right) = \frac{\exp\left(h_l\left(\sigma_l^{(m)}\right) + \sum_{k=1,k\neq l}^{L} J_{lk}\left(\sigma_l^{(m)}, \sigma_k^{(m)}\right)\right)}{\sum_{q=1}^{21} \exp\left(h_l(q) + \sum_{k=1,k\neq l}^{L} J_{lk}\left(q, \sigma_k^{(m)}\right)\right)}$$

This equation can be interpreted as a Multinomial logistic regression model, a "log-linear" model. The outcome term is the l-th position, and the input features are other positions except l-th position. $J_{lk}(a,b)$ can be considered as a regression coefficient associated with the residue type b at position k variable and the outcome (l-th position) with residue type b. $h_l(b)$ can be interpreted as the bias parameter of position l being residue type b.

In addition, the inverse of covariance matrix (precision matrix) can also be interpreted as linear regression models [1,2].

References

1. Kwan CC. A regression-based interpretation of the inverse of the sample covariance matrix. Spreadsheets in Education. 2014;7(1):4613.
2. Stevens GV. On the inverse of the covariance matrix in portfolio analysis. The Journal of Finance. 1998;53(5):1821-7.
