# Supplementary material for: Deducing high-accuracy protein contact-maps from a triplet of coevolutionary matrices through deep residual convolutional networks
Source: PLoS Comput Biol. 2021 Mar 26;17(3):e1008865. doi: 10.1371/journal.pcbi.1008865 (PMC8026059; doi:10.1371/journal.pcbi.1008865)
Supplement: S2 Text — (PDF) [file pcbi.1008865.s007.pdf]

S2 Text. Detailed procedure to collect training and test datasets

50 CASP FM targets and 195 CAMEO hard targets. The 50 non-redundant FM domains from the CASP11 and CASP12 experiments are downloaded from http://predictioncenter.org/download_area/. The 195 non-redundant targets defined as *hard* by CAMEO are collected from https://www.cameo3d.org/sp/targets/1-year/?to_date=2019-07-20 whose Submission Date is from 2018-07-28 to 2019-07-20. Both of the two test datasets have a pair-wise sequence identity < 30% within each of the two datasets. All sequences share >30% to any proteins used in training the TripletRes models are removed. For a fair comparison, those targets in test sets were also removed if they cannot be finished in 72 hours by any of the control methods.

7,671 SCOPe 2.07 domain sequences. TripletRes was trained on a subset of SCOPe 2.07 (downloaded in March 2018) domain sequences collected as per the following criteria: (1) Sequence length should be in the range of 30-400 residues; (2) Resolution of the corresponding structure should be better than 2.0 Å; (3) Maximum pairwise sequence identity is also set to 30%. There were 7,671 domains collected for training. The whole training set was split into 10 subsets, and we randomly selected one as the validation set and left the remaining subsets as the training set for hyper-parameter tuning. After the hyper-parameter tuning, the final model is the average of 10 models and each model was trained by considering each subset as the validation set and the remaining subsets as the training set.

26,151 PDB sequences. A new training set was constructed to train TripletRes, which is a non-redundant set of experimental structures from Protein Data Bank (PDB). A total number of 510,940 structures by 2019.11.12 with a maximum length of 1000 residues were initially collected. The initial sequence set was then clustered at the sequence identity threshold of 35%. The obtained 26,151 full-length sequence representatives are selected as the final training set.

37 hybrid test sequences. To objectively evaluate the performance of TripletRes trained with the new training data, a test set containing 37 sequences was constructed. The test set was the combination of 50 non-redundant FM domains from the CASP11 and CASP12 experiments, 195 non-redundant targets defined as *hard* by CAMEO and 31 FM domains in CASP13. We excluded those sequences that have a sequence identity > 40% with any sequence in the training set.
