# Supplementary material for: Deducing high-accuracy protein contact-maps from a triplet of coevolutionary matrices through deep residual convolutional networks
Source: PLoS Comput Biol. 2021 Mar 26;17(3):e1008865. doi: 10.1371/journal.pcbi.1008865 (PMC8026059; doi:10.1371/journal.pcbi.1008865)
Supplement: S3 Text — (PDF) [file pcbi.1008865.s008.pdf]

S3 Text. A brief introduction of control methods and other top participants in CASP13

CCMpred [1] is a representative DCA method, and the several of other control methods, DNCON2 [2], MetaPSICOV2 [3], DeepContact [4], and ResPRE [5], are based on supervised machine learning models using outputs of CCMpred or other DCA methods as input feature. Here, DNCON2, MetaPSICOV2 and DeepContact are the top-ranking predictors in CASP12 and ranked closely to the best method, RaptorX-Contact, in CASP12 [6]. ResPRE was our previous work which was built on raw precision matrix feature and shown to be comparable with many state-of-the-art methods despite the use of a single precision feature matrix. It should be noted that CCMpred does not have a built-in program for MSA generation. For a fair comparison, we tested it with the same MSAs as those used in the test phase of TripletRes. The control methods were downloaded and implemented in our local computers with default parameters and the versions of sequence databases are identical to those of DeepMSA.

In CASP13, DMP, also known as DeepMetaPSICOV [7], combines the input features of MetaPSICOV2 and a covariance feature [8] with residual convolutional neural networks (RCNNs). Meanwhile, both ZHOU-Contact, i.e. SPOT-Contact [9], and RaptorX-Contact [10] combine traditional one-dimensional features (secondary structure, solvent accessibility, and sequence profile, etc.) and pairwise coevolution features (CCMpred final output) by RCNNs or recurrent neural networks. The prediction results of other participants in CASP13 were obtained from CASP13 data archive.

References

1. Seemayer S, Gruber M, Söding J. CCMpred—fast and precise prediction of protein residue–residue contacts from correlated mutations. Bioinformatics. 2014;30(21):3128-30.
2. Adhikari B, Hou J, Cheng J. DNCON2: improved protein contact prediction using two-level deep convolutional neural networks. Bioinformatics. 2017;34(9):1466-72.
3. Buchan DW, Jones DT. Improved protein contact predictions with the MetaPSICOV2 server in CASP12. Proteins: Structure, Function, and Bioinformatics. 2017.
4. Liu Y, Palmedo P, Ye Q, Berger B, Peng J. Enhancing evolutionary couplings with deep convolutional neural networks. Cell systems. 2018;6(1):65-74. e3.
5. Li Y, Hu J, Zhang C, Yu DJ, Zhang Y. ResPRE: high-accuracy protein contact prediction by coupling precision matrix with deep residual neural networks. Bioinformatics. 2019:4647-55.
6. Schaarschmidt J, Monastyrskyy B, Kryshtafovych A, Bonvin AMJJ. Assessment of contact predictions in CASP12: Co-evolution and deep learning coming of age. Proteins: Structure, Function, and Bioinformatics. 2018;86(S1):51-66.
7. Kosciolek T, Jones DT. Accurate contact predictions using covariation techniques and machine learning. Proteins: Structure, Function, and Bioinformatics. 2016;84(S1):145-51.
8. Jones DT, Kandathil SM. High precision in protein contact prediction using fully convolutional neural networks and minimal sequence features. Bioinformatics. 2018;34(19):3308-15.

9. Hanson J, Paliwal K, Litfin T, Yang Y, Zhou Y. Accurate prediction of protein contact maps by coupling residual two-dimensional bidirectional long short-term memory with convolutional neural networks. Bioinformatics. 2018;34(23):4039-45.
10. Xu J. Distance-based protein folding powered by deep learning. Proc Natl Acad Sci U S A. 2019;116(34):16856-65.
