# Supplementary material for: Deducing high-accuracy protein contact-maps from a triplet of coevolutionary matrices through deep residual convolutional networks
Source: PLoS Comput Biol. 2021 Mar 26;17(3):e1008865. doi: 10.1371/journal.pcbi.1008865 (PMC8026059; doi:10.1371/journal.pcbi.1008865)
Supplement: S4 Text — (PDF) [file pcbi.1008865.s009.pdf]

S4 Text. Traditional feature extraction strategy with post-processing

As a baseline for comparison, the traditional feature extraction method, which involves a post-processing procedure over a raw coevolutionary feature matrix, is tested. There are usually two steps in the post-processing procedure. The coevolutionary feature matrix is first transformed into an *L* by *L* contact score matrix *C* by

$$C_{ij} = \sqrt{\sum_{a,b} \left\| F_{ij}^{ab} \right\|_2^2} \tag{A}$$

where each entry represents the potential of forming a contact. Here, *a* and *b* represent two types of amino acids, and *F* represents the obtained coevolutionary feature matrix. The contact score matrix *C* will be further normalized by an average product correction (APC) step:

$$C_{ij}^{APC} = C_{ij} - \frac{C_i C_j}{C} \tag{B}$$

where $C_i = \frac{1}{L}\sum_{j \neq i}^{L} C_{ij}$, and $\mathrm{C} = \frac{1}{L^2 - L}\sum_{i,j,i<j}^{L} C_{ij}$. C^{APC} is the predicted contact-map based on coevolution analysis with the post-processing procedure. C^{APC} can be considered as the input feature of a supervised machine learning model. In this work, we use the same neural network structure with 22 residual blocks as the supervised learning model for the comparison of the two extraction strategies.
