# Supplementary material for: Deducing high-accuracy protein contact-maps from a triplet of coevolutionary matrices through deep residual convolutional networks
Source: PLoS Comput Biol. 2021 Mar 26;17(3):e1008865. doi: 10.1371/journal.pcbi.1008865 (PMC8026059; doi:10.1371/journal.pcbi.1008865)
Supplement: S5 Text — (PDF) [file pcbi.1008865.s010.pdf]

S5 Text. Binary cross entropy loss function for training TripletRes in CASP13.

The loss function is defined as the sum of cross entropy over all the residue pairs of the training proteins:

$$\mathcal{L}_{bin} = -\sum_{t=1}^{T} y_t \log(p_t) + (1 - y_t) \log(1 - p_t) \qquad \text{(A)}$$

Here, T is the total number of residue pairs in the training set. $y_t = 1$ if the distance of t-th residue pair of native structure is below 8Å; otherwise $y_t = 0$. p_t is the predicted probability that the t-th residue pair forms a contact.
